# B7-H4 overexpression is essential for early hepatocellular carcinoma progression and recurrence

**DOI:** 10.18632/oncotarget.20718

**Published:** 2017-09-08

**Authors:** Fu-Biao Kang, Ling Wang, Dian-Xing Sun, Hai-Jun Li, Dong Li, Yan Wang, Ji-Wen Kang

**Affiliations:** ^1^ The Liver Disease Diagnosis and Treatment Center, Bethune International Peace Hospital, Shijiazhuang, Hebei, P.R. China; ^2^ Department of Orthopedic Oncology, The Third Hospital of Hebei Medical University, Shijiazhuang, Hebei, P.R. China

**Keywords:** hepatocellular carcinoma, B7-H4, cancer progression, cancer recurrence

## Abstract

B7-H4, another member of costimulatory molecule, has been shown to be overexpressed in multiple types of tumors, including hepatocellular carcinoma (HCC). However, the specific biological role of B7-H4 in HCC still needs to be further explored. In this study, we observed that B7-H4 was highly overexpressed in HCC tissues and cells, and its overexpression strongly correlated with patient's TNM stage, overall survival and early recurrence. Downregulation of B7-H4 significantly suppressed cell growth, invasion, and stemness of HCC by inducing apoptosis in the *in vitro* experiment. In addition, depletion of B7-H4 could help restore CD8^+^ T anti-tumor immunity by elevating the expression and secretion levels of CD107a, granzyme A, granzyme B, perforin and IFN-γ. In a xenografted mouse model of HCC, stable depletion of B7-H4 resulted in significantly smaller mean tumor volume and less mean tumor weight after 30 days of growth, compared to the control group. Together, our results provide insights into the diverse functions of B7-H4 involved in the pathogenesis, recurrence and anti-tumor immunity of HCC, indicating B7-H4 as a novel and effective approach for future treatment strategies that benefits anticancer therapy.

## INTRODUCTION

Hepatocellular carcinoma (HCC) is the sixth most common malignant tumor, and has moved up the rank from the third most common cause of cancer death to the second according to the recently released World Cancer Report 2014 [1]. In China, HBV and HCV are major responsible for HCC, which is the leading cause of cancer death in male patients within 60 years old [2, 3]. Majority of the HCC patients will probably die within 9-12 months after diagnosis, which is due to being resistant to conventional chemotherapeutic agents and high recurrence and metastasis after resection [4, 5]. Despite many attempts to develop molecularly-targeted agents and immunotherapy strategies, sorafenib remains the only currently FDA-approved multi-kinase inhibitory agent for the systemic therapy of advanced HCC. However, sorafenib could expand the survival only 2.8 months [6, 7]. Therefore, therapeutic targets of most novel agents are unmet needs in HCC.

B7-H4, also known as VTCN1, B7x or B7S1, is an important member of the B7 family co-regulatory ligands [8–10]. B7-H4 could negatively regulate T cell-mediated immunity by controlling cytokine secretion, cytotoxicity development and activation of T cells [11, 12]. B7-H4 cell surface protein expression is largely absent in most normal human somatic tissues, whereas overexpressed in a variety of cancers including lung, ovarian, breast, uterus, prostate, renal, gastric, pancreatic ductal adenocarcinoma and esophageal cancers [13–17]. Studies have shown that B7-H4 could be a crucial driver in the development and progression of human carcinomas by binding to an unknown receptor [18]. Currently, there is only some evidence showing that serum B7-H4 seems to be a promising biomarker and a candidate therapeutic target for HCC, but the potential mechanism still remains elusive [19, 20].

Here, the expression of B7-H4 was studied on patient HCC samples with early recurrence (recurrent HCC disease within two years) or non-recurrence (no recurrent disease after five years). Next, we employed RNAi gene silencing technology to investigate the functional and signaling roles of B7-H4 in HCC growth and progression, as well as underlying molecular mechanisms. To complement these *in vitro* studies, we examined the effects of B7-H4 depletion on tumorigenicity in nude mice. Results from our *in vitro* and *in vivo* analyses demonstrated that B7-H4 would be a valuable biomarker and promising therapeutic target for HCC in humans.

## RESULTS

### B7-H4 is highly overexpressed and strongly associated with poor prognosis and early recurrence in HCC

**Figure 1:**
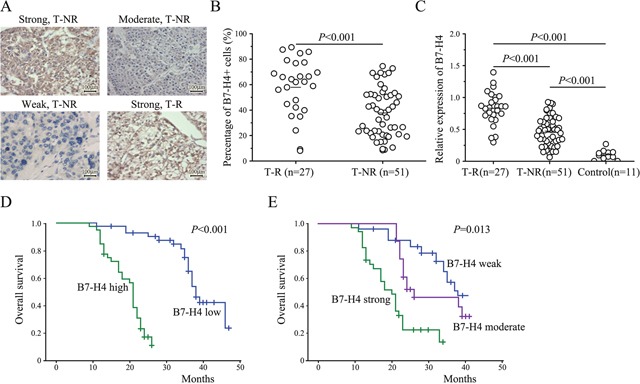
The expression of B7-H4 in HCC and the overall survival of 78 HCC patients stratified by B7-H4 protein expression and intensity levels **(A)** The representative IHC staining results for validation of B7-H4 expression in HCC tumor tissues with (T-R) or without early recurrence (T-NR) (200×, scale bar: 100μm). **(B)** The imaging analysis and quantification of IHC staining with B7-H4. **(C)** The expression of B7-H4 by RT-qPCR in 27 T-R, 51T-NR, and 11 cases of normal liver tissues. **(D)** Kaplan-Meier overall survival curve of HCC patients in correlation with B7-H4 expression level (n=185). **(E)** Kaplan-Meier overall survival curve of HCC patients in correlation with B7-H4 intensity level (n=185).

To identify the clinical importance of B7-H4 in HCC, we analyzed the expression of B7-H4 in paraffin–embedded tumors and adjacent non-tumor tissues (NAT) of 78 HCC patients by IHC staining. Results revealed that B7-H4 protein expression was significantly increased in HCC tumors and that the immunolocalization of B7-H4 molecules was predominantly in the membrane and cytoplasm of liver tumor cells (Figure 1A). In comparison, more significant increase in the intensity of tumor B7-H4 staining was observed in early-recurrence HCC tumors (Figure 1B). These results were further validated by qRT-PCR analysis in an independent cohort of HCC samples, which included 27 cases of tumor-recurrence patients, and 51 cases of tumor non-recurrence patients, and 11 cases of normal liver tissues (Figure 1C). The association between B7-H4 expression level and the clinical pathological characteristics was shown in Table 1. Results showed that B7-H4 expression was associated with vascular invasion, TNM stage, and lymph node metastasis (p=0.003, p=0.031, and p=0.038). Kaplane-Meier analysis showed that high expression of B7-H4 in HCC tissues and was significantly associated with shorter OS rate in HCC patients (Figure 1D). Cases with strong or moderate B7-H4 staining intensity in HCC are prone to have a significantly shortened mean survival time compared to the cases with weaker staining (log-rank test, p=0.015, Figure 1E). According to the univariate and final multivariate Cox regression analysis, B7-H4 staining intensity was an independent prognostic factor for OS in HCC patients (RR: 0.256, 95% CI: 0.171–0.901, p=0.019, Supplementary Table 1). Therefore, these data suggest that weaker B7-H4 expression was correlated with better prognosis or survival in HCC.

**Table 1:** Relationship between B7-H4 expression and clinico-pathological parameters in HCC

Index	Case	B7-H4 expression		*p*
		Low	High	
**Age(y)**				
≤50	36	9	27	0.452
>50	42	15	37	
**Gender**				
Male	59	18	41	0.317
Female	19	7	12	
**Vascular Invasion**				
+	37	5	32	**0.003**
-	41	16	25	
**TNM stage**				
I-II	49	21	28	**0.031**
III-IV	29	5	24	
**Lymph metastasis**				
+	22	2	20	**0.038**
-	56	17	39	
**Tumor size(cm)**				
≤5	35	11	24	0.052
>5	43	8	35	
**Tumor encapsulation**				
+	18	3	15	0.041
-	60	21	39	
**Child-Pugh classification**				
A	37	12	25	0.185
B	41	13	28	
**Liver cirrhosis**				
weak	19	8	13	0.173
moderate	38	11	27	
strong	21	6	15	
**HBsAg**				
+	48	12	36	0.211
-	31	9	22	
**AFP**				
≤200	40	19	21	0.192
>200	38	8	30	
**Transfusion**				
+	36	9	27	0.306
-	42	19	23	

Values in bold signify *p*<0.05.

### Downregulation of B7-H4 inhibits cell viability and induces apoptosis in HCC

**Figure 2:**
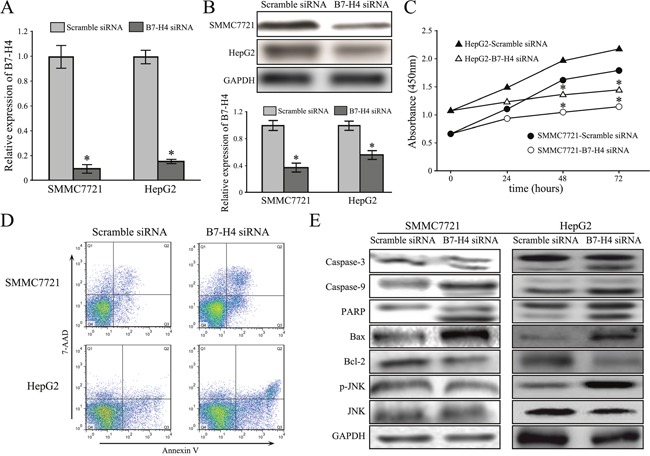
Downregulation of B7-H4 suppresses cell growth and apoptosis in HCC cells **(A-B)** The knockdown effects of B7-H4 in SMMC7721 and HepG2 cells by B7-H4 via RT-qPCR and western blot. **(C)** The relative cell viability of SMMC7721 and HepG2 cells at 24-72 h after B7-H4 downregulation. **(D)** The representative images showed that the increase apoptotic cells were observed by flow cytometry analysis in both SMMC7721 and HepG2 cells with siB7-H4 transfection. **(E)** B7-H4 RNAi influenced the expression of molecules associated with apoptosis via JUK activation. Data was presented as mean ± SD; *p<0.05.

To decipher the biological function of B7-H4 in HCC, we performed transient knockdown via RNA interfering technology targeting B7-H4 and examined by RT-qPCR and western blot analysis. Among the three pairs of oligonucleotides that target B7-H4, two of them effectively decreased the B7-H4 mRNA and protein expression in HCC cells. We selected the best one to carry out further experiments (Figure 2A-2B). CCK-8 assay was used to evaluate the potential effect of B7-H4 downregulation on HCC cell growth. Knockdown of B7-H4 significantly reduced SMMC7721 and HepG2 cell viability after 48 h of transient transfection (Figure 2C). Moreover, transient knockdown of B7-H4 in SMMC7721 and HepG2 cells significantly increased the detectable Annexin V-positive apoptotic cells after 48 h of transient transfection by using Annexin V and 7-ADD double staining (Figure 2D). Western blot analysis was performed to evaluate the expression of apoptotic molecules. We observed the increase of pro-apoptotic proteins, Bax and the cleaved caspase-3 and caspase-8, PARP, and together with decrease of anti-apoptotic protein, Bcl-2 relative to their controls. Furthermore, knockdown of B7-H4 increased JNK phosphorylation, but did not change total JNK protein expression level in HCC cells (Figure 2E).

### Downregulation of B7-H4 inhibits cell invasion and stemness characteristic in HCC

**Figure 3:**
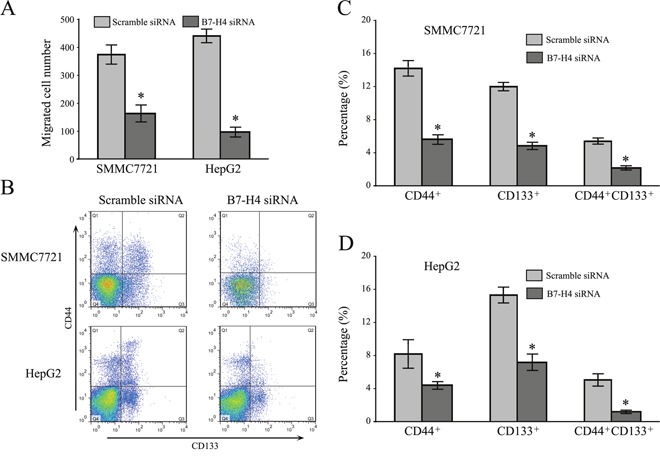
Downregulation of B7-H4 suppresses cell invasion and stemness in HCC cells **(A)** The knockdown effects of B7-H4 on cell invasion of SMMC7721 and HepG2 cells by transwell chamber assay. **(B)** The effects of B7-H4 knockdown on the expression of cancer stem-like marker, CD44 and CD133, on the surface of SMMC7721 and HepG2 cells. Data was presented as mean ± SD; *p<0.05.

To address the role of B7-H4 siRNA in the aggressive behaviors of HCC cells, transwell assay was performed to evaluate the influence of B7-H4 siRNA on migration and invasion. As shown in Figure 3A, the average transmembrane cells that depleted B7-H4 (179.3±18.1 for SMMC7721 cells, 95.8±11.6 for HepG2 cells, *p*<0.05) were significantly less than these of the control group (381.6±19.8 for SMMC7721 cells, 442.8±27.1 for HepG2 cells, *p*<0.05). These results suggested that the abilities of migration and invasion were significantly inhibited in SMMC7721 and HepG2 cells after B7-H4 downregulation, compared to that in control cells, respectively. These data indicate that B7-H4 plays curtail roles in the motility and promoted invasion of HCC cells.

Cancer stem cells (CSCs) are thought to contribute to tumor recurrence and metastasis. CD44 and CD133 have been reported as potential biomarkers of HCC. Here, flow cytometry analysis demonstrated that SMMC7721 and HepG2 cells gave a decreased in the CD44^+^/CD133^+^ double positive cell subpopulation after B7-H4 downregulation (5.68±0.78 vs 1.89±0.12 for SMMC7721 cells; 5.13 ±0.89 vs 0.98±0.07 for HepG2 cells, Figure 3B-3D) in SMMC7721 and HepG2 cells. Results provided corroborative evidence for silencing B7-H4, which reduced the stemness property of HCC cells.

### Depletion of B7-H4 expression promotes CD8^+^ T cell-mediated cytotoxicity

**Figure 4:**
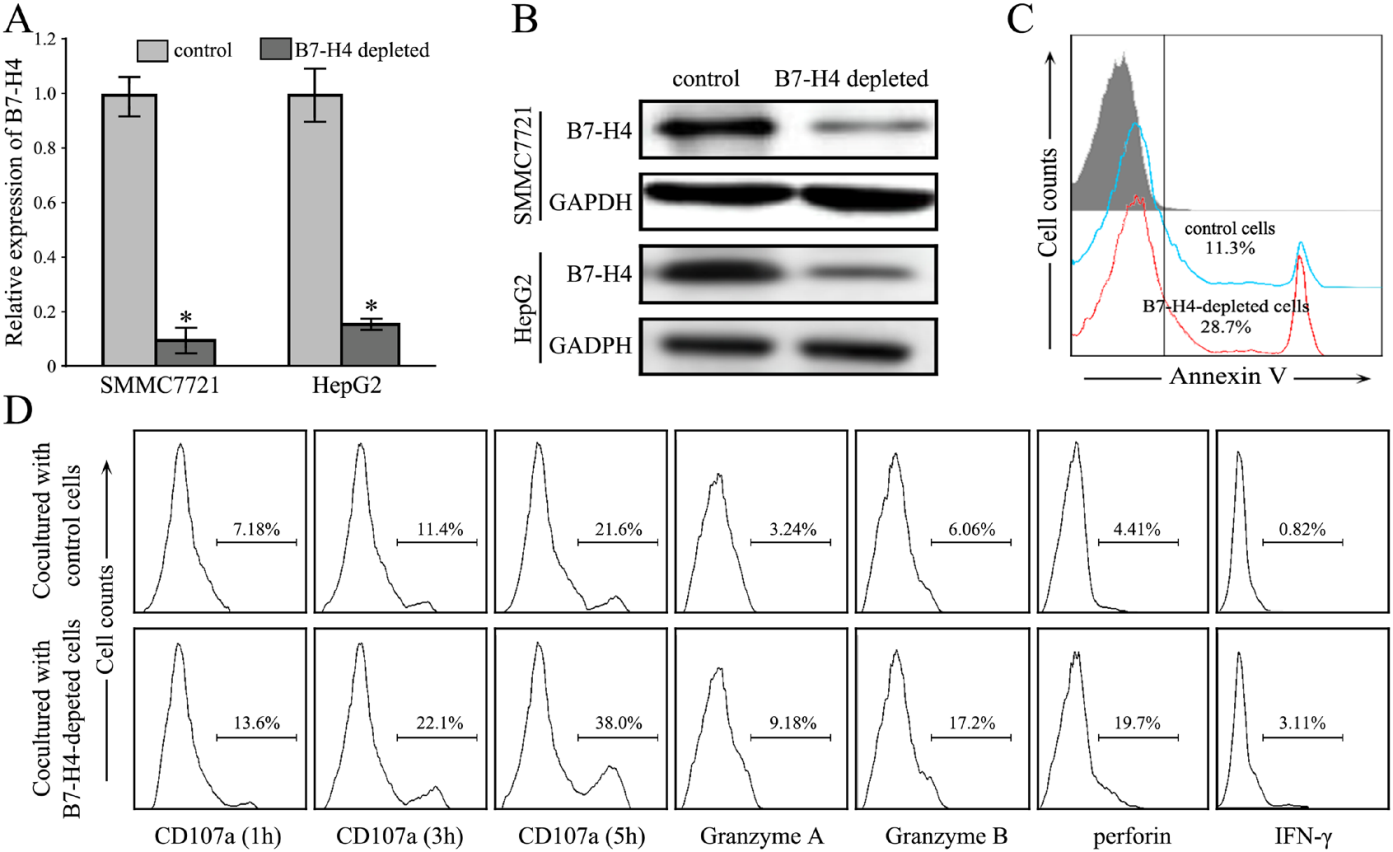
Depletion of B7-H4 restores CD8+ T cells anti-tumor immunity **(A-B)** The stable knockdown effect of B7-H4 in SMMC7721 cells was evaluated by RT-qPCR analysis and by western blotting analysis. **(C)** The representative apoptotic rate of SMMC7721 cells depleted B7-H4 after culturing with allogeneic CD8^+^ T cells *in vitro*, compared with wild type SMMC7721 cells. **(D)** The representative cytokine profile of CD8^+^ T cells was determined by flow cytometry analysis after culturing with SMMC7721 cells depleted B7-H4 and wild type SMMC7721 cells. Data was presented as mean ± SD; *p<0.05.

To study the function of B7-H4 in tumor-associated immune cells, we generated stable knockdown cells of B7-H4 by transfecting into SMMC7721 cells and HepG2 cells (Figure 4A-4B). Cell apoptosis assay in the coculture system, isolated allogeneic CD8^+^ T cells from normal individuals and were incubated with B7-H4-depleted SMMC7721 cells or wild-type control cells in the presence of anti-CD3 antibody and rhIL-2. The apoptotic rate of depleted B7-H4 HCC cells was significantly increased when cocultured with isolated CD8^+^ T cells, when compared to wild-type SMMC7721 cells (*p*=0.027, *n*=5) (Figure 4C). Furthermore, when the depleted B7-H4 HCC cells were cocultured with allogeneic PBMCs, the blockade of B7-H4 increased the expression of CD8^+^ T cell effector cytokines Granzyme A (*p*=0.023), Granzyme B (*p*=0.006), perforin (*p*=0.030) and IFN-γ (*p*=0.011) (*n*=5, Figure 4D). Especially, the depletion of B7-H4 resulted in a significant restoration of CD107a expression at 1, 3, and 5 hours in the coculture system (respectively *p*=0.002, *p*=0.005, and *p*=0.002, *n*=5). These findings implied that over-expression of B7-H4 in HCC cells impaired CD8^+^ T cell-mediated cytotoxicity.

### Depletion of B7-H4 expression inhibits tumorigenicity in HCC

**Figure 5:**
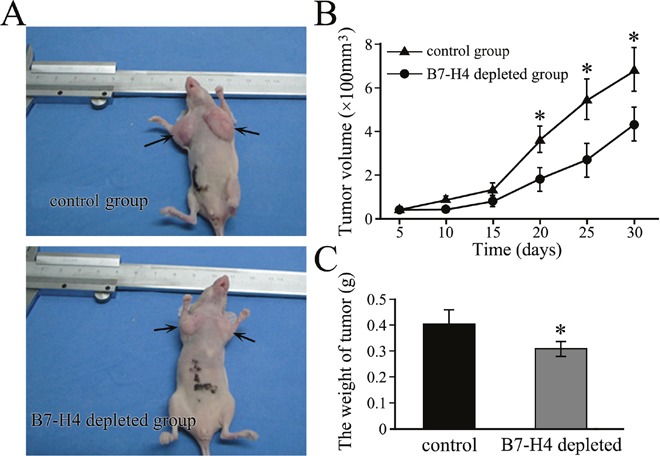
Depletion of B7-H4 inhibits the growth of subcutaneous xenograft tumors in nude mice **(A)** Representative xenograft tumors are grown for 10 days using SMMC7721 cells by stable knockdown of B7-H4 or wild-type control cells. The tumors are shown by arrows. **(B)** The tumor volume and **(C)** tumor weight were compared between the stable cells and control cells. *p<0.05, compared with control.

To confirm the function of B7-H4 in HCC *in vivo*, BALB/C nude mice were inoculated with negative control SMMC7721 cells or those stably transfected with B7-H4 shRNA. The representative expressions of B7-H4 protein in xenografts of each group showed in Supplementary Figure 1. Tumors were visible after 7 days in both the groups of mice groups, but tumors from B7-H4-shRNA cells grew showed a significantly slower growth (Figure 5A). Mean tumor volume at the end of the 30-day observation period was 656.19 ± 21.08 mm^3^ in the negative control group and 311.79 ± 19.35 mm^3^ in the B7-H4-shRNA cells group (*p*= 0.028), while the corresponding mean weights were 0.418 ± 0.051g and 0.276 ± 0.012 g (*p*=0.011, Figure 5B-5C), respectively. Our results indicated that B7-H4 gene depleting depletion led to the inhibition of tumor growth in HCC.

## DISCUSSION

HCC is the common malignancy with the most common leading cause of cancer-associated mortality globally, which contributes to the poor prognosis and early intrahepatic and extrahepatic recurrence in HCC patients [21, 22]. Although surgical resection accompanied by liver transplantation provides some hope to patients with early stage HCC, the OS of HCC patients is still far from satisfying [23]. Recently, immunotherapy has become an important and effective complementary approach to conventional HCC treatments [24, 25]. Immune checkpoint blockade therapy represents a major breakthrough in cancer treatment and promising results have been presented at the 2015 American Society of Clinical Oncology (ASCO) annual meeting from a phase I/II trial of the anti-PD-1 antibody nivolumab in HCC patients [6, 26, 27]. Here, another immune-mediated molecule, B7-H4 is a newly described member of the B7/CD28 family, and has been suggested to have strong immunoinhibitory properties in tumors. To date, B7-H4 gene has been intensively studied in numerous tumors [28, 29]. Previous studies have shown that high B7-H4 expression was significantly correlated with the size of tumor, depth of tumor infiltration, survival time, metastasis and recurrence [11, 30, 31]. Moreover, the level of B7-H4 in serum was also evaluated in various cancer patient populations, including HCC [19, 20]. Based on the comprehensive evidence, the investigation of B7-H4 expression and its function in HCC provides a potential target for novel immunotherapeutic approaches.

According to our investigation about the presence and functional significance of B7-H4 expression in HCC, strong B7-H4 positivity in HCC that is correlated with tumor TNM stage, vascular invasion, and lymph node metastasis existed, which was consistent with the expression pattern of B7-H4 in other tumors [13, 32]. These results predicted that B7-H4 might play a role in the development of HCC. To further study regarding the role of B7-H4 in tumor progression, we investigated the effects of B7-H4 expression on the biological functions of SMMC7721 and HepG2 cells *in vitro*. Park et al showed that B7-H4 could significantly reduce the cell growth of Raji and IM-9 cells via arresting G0/G1 phase of the cell cycle [33]. Our results showed that proliferation of SMMC7721 and HepG2 cells were significantly suppressed after transient knockdown of B7-H4. Downregulation of apoptotic pathways is a hallmark of cancer. Bcl-2 is a key regulator of the intrinsic pathway and acts by preventing the release of pro-apoptotic molecules from mitochondria into the cytosol, while Bax promotes apoptosis by inducing mitochondrial outer membrane permeabilization [34, 35]. In this study, SMMC7721 and HepG2 cells were transfected with B7-H4-siRNA, which resulted in the decrease of anti-apoptotic protein, Bcl-2 and increase of the pro-apoptotic protein, Bax, leading to further cleavage of caspase-3 and -8, probably via activation of JNK pathway. But the link between B7-H4 and JNK signaling pathway needs to be further investigated. Moreover, silencing of B7-H4 could induce apoptosis and inhibit cell invasion and stemness of HCC cells. HCC stem cells possess the capacity for self-renewal, differentiation, and responsible for tumor initiation, proliferation and progression. CD44 and CD133 were highly enriched with properties of HCC stem cells [36, 37]. Importantly, depletion of B7-H4 led to the decreased CD44^+^/CD133^+^ double positive cell subpopulation, suggesting that the overexpression of B7-H4 in HCC cells might be potentially associated with its stemness properties. Many studies have revealed that the cancer stem cells may be present as a distinctly small population driving relapse and metastasis by giving rise to new chemoresistant tumors. If B7-H4 could decrease the formation ratio of HCC stem cells, which meant that it could stop further metastasis in HCC patients. With its application clinically, B7-H4-based agents have the advantage of recognizing a small population of cancer stem cells that has a severe malignancy and offers as perfect treatment targets. Although B7-H4 inhibition might offer a promising opportunity to inhibit the progression and metastasis of human HCC, more work should be performed in detail to further test the mechanisms of B7-H4 in anti-HCC stemness and invasion.

B7-H4 was identified as a negative co-stimulatory molecule via down-regulating immune responses by reducing T cell proliferation and cytokine production. Sica et al showed that blockade of endogenous B7-H4 by a specific mAb promoted T-cell response [8, 38]. These study results showed that putative receptor of B7-H4 could be upregulated on activated T cells, exerting a profound inhibitory effect on the growth, cytokine secretion, and development of cytotoxicity. In our study, depleted B7-H4 induced the apoptotic rate of HCC cells encountered with CD8^+^ T cells, and consequently increased the expression of CD8^+^ T cell effector cytokines Granzyme A, Granzyme B and IFN-γ. CD107a is a lysosomal-associated membrane glycoprotein that surrounds the core of the lytic granules in cytotoxic T cells. Our study showed that CD8^+^ cytotoxic T lymphocytes (CTLs) from HCC patients showed significantly lower levels of exocytosis of cytolytic molecule, CD107a, in response to TCR engagement; however, it could be upregulated after B7-H4 downregulation. Therefore, it is proposed that chimeric antigen receptor T-cell approaches that directly target B7-H4 might be effective by simultaneously destroying B7-H4^+^ tumor cells and eliminating negative-immune modulating cells from the tumor microenvironment. In addition, the B7-H4 functional studies were carried out with human HCC cell line xenografts in SCID mice, but lacked most of the elements of a functional immune system. Two groups of xenograft growth provide direct evidences that B7-H4 gene depletion resulted in the obvious variation of tumor volume and weight. Therefore, our data indicated that B7-H4 promoted tumor cell growth, which revealed a new tumor-specific function of this protein in modulating the immune cell function. It seems that the inhibitory effects of B7-H4 on T cells are not the same as those participating in the tumor promoting effects of B7-H4 in epithelial cancer cells.

In summary, the clinical data indicated that higher expression of B7-H4 correlated with vascular invasion and reduced recurrence-free survival, suggesting the involvement of B7-H4 activation in promoting HCC recurrence. *in vivo* and *in vitro* results demonstrated that B7-H4 was involved in the regulation of biological characteristics of HCC, including anti-apoptosis, invasion, migration, stemness and even accelerate the xenografted tumor growth. These data imply that B7-H4 might be a novel biomarker for predicting the prognosis and recurrence, and act as a potential therapeutic target in the treatment of early HCC.

## MATERIALS AND METHODS

### Patients and specimens

Fresh HCC tissues and surrounding adjacent non-tumor liver tissues (at least 3 cm distant from the tumor site) were obtained from 78 patients with pathologically confirmed HCC. None of the patients had received anticancer therapy before surgical resection, and patients with concurrent autoimmune disease, HIV, or syphilis were excluded. Clinical stages were classified according to the International Union against Cancer. An additional 107 HCC patients who had undergone curative resection between 2002 and 2007 and had complete follow-up data were enrolled for analysis of overall survival (OS). The research was approved by the Institutional Review Board of Bethune International Peace Hospital. Both written and oral consent was obtained before the samples were collected from the participating patients, and relevant clinical and histopathological data provided to the researchers were anonymized.

### Cell lines and culture conditions

Human HCC cell lines (SMCC-7721 and HepG2) were bought from the Cell Bank of the Chinese Academy of Sciences (Shanghai, China). Cells were cultured in Dulbecco's modified Eagle's medium (DMEM) with 10% FBS and 100 units/mL of penicillin and 100 mg/mL of streptomycin (Invitrogen, Carlsbad, CA). The cells were maintained in high-glucose DMEM (Gibco) supplemented with 10% fetal calf serum (Gibco). The cells were then incubated at 37°C in a humidified chamber containing 5% CO2.

### Immunohistochemical staining

The paraffin-embedded samples were cut into 5-μm sections using the DakoEnVision^TM^ method according to the manufacturer's instructions. After deparaffinization in xylene, the sections were cooled down and immersed in 0.3% H_2_O_2_ solution for 20 min to block endogenous peroxidase activity, and then rinsed in PBS for 5 min, blocked with 5% BSA at room temperature for 20 min, and incubated with primary antibodies against B7-H4 (ab209242, Abcam Company, 1:300 dilution) at 4°C for overnight. Negative controls were performed by replacing the specific primary antibody with PBS. After washing three times with PBS, sections were incubated with secondary antibodies for 30 min at room temperature. Diaminobenzene was used as the chromogen and hematoxylin as the nuclear counterstain. Sections were dehydrated, cleared and mounted. The slides were viewed and imaged with a microscope system (Olympus). All slides were reviewed by two independent hematopathologists who were blinded to the clinical outcomes. Evaluation of B7-H4 staining in cancer cells was assessed by authorized pathologists who had no knowledge of the patients’ clinical status. Quantification was made as follows; ≤33% of the cancer cells: 1, >33 to ≤66% of the cancer cells: 2, >66% of the cancer cells: 3; intensity of staining: absent/weak: 1, moderate: 2, strong: 3. The intensity of B7-H4 staining was considered weak when either cytoplasmic expression or rare membranous condensation was present, moderate when incomplete and discontinuous moderate membranous expression was present, and strong when complete membranous expression of the molecule was present. Each section had a final grade that derived from the multiplication of the area and intensity scores. Sections with a final score of ≤3 were classified as tumors with low B7-H4 expression, whereas sections with a final score of >3 were classified as tumors with high B7-H4 expression.

### B7-H4 siRNA transfection and stable cell line construction

To further analyze the role of B7-H4 in HCC malignancy, SMMC7721 and HepG2 cells were transfected with B7-H4 siRNA plasmid purchased from Origene Technologies Inc using Lipofectamine^3000^. Plasmid DNA dilution and liposome diluent were mixed, incubated and then transfected into the cells in a step-wise manner according to the manufacturer's instructions. In order to establish the stable B7-H4 depleted HCC cells, recombinant lentiviral vector mediated shRNA-targeted B7-H4 was constructed and by observing the expression of GFP. SMMC7721 and HepG2 cells were seeded into six-well plates and allowed to grow at 70–80% confluence. Then, the cells were infected with retroviral particles (negative control) or containing B7-H4 shRNA in the presence of polybrene and incubated for 24 h at 37°C. The medium was replaced with DMEM containing 10% FBS and 1 μg/ml puromycin without other antibiotics for 2 weeks.

### Quantitative real-time reverse transcription-PCR (RT-PCR)

Total RNA was extracted from the cells using Trizol (Invitrogen) according to the manufacturer's protocol. cDNA was synthesized using an M-MuLV cDNA Synthesis kit as per the manufacturer's instructions (Thermo Scientific), followed by quantitative PCR in a 7500 Real-Time PCR System using SYBR® Premix Ex Taq^™^ II (Takara Biotechnology). Primer sets were designed for human B7-H4 as: (forward) 5′-TCCTCCAGAAAAGCACAAGGAT-3′; (reverse) 5′-CATCCTCCCCAATGTTCCCA -3′. Standard curves were established for each primer set and both reference and target reactions were performed for each sample. All results were calculated using the 2^[−ΔΔC (T)]^ method. All experiments were performed in triplicates.

### Western blot analysis

Protein lysates from cultured cells were obtained using RIPA buffer. About 20 μg protein was extracted from each sample and resolved using 8%, 10% or 12% SDS-polyacrylamide gel electrophoresis, transferred to a nitrocellulose membrane, blocked in 5% nonfat milk and blotted with an appropriate antibody. Rabbit anti-B7-H4 and mouse anti-Bcl-2, Bax, caspase-3, caspase-8 and PARP antibodies were purchased from Abcam Company. Rabbit anti-phospho-JNK and JNK antibodies were purchased from Cell Signaling Technology. Anti-β-actin antibody was purchased from Santa Cruz Biotechnology, and then incubated with anti-rabbit or anti-mouse IgG and detected using enhanced chemiluminescence reagents (Thermo Fisher Scientific).

### Flow cytometric analysis

FITC-conjugated anti-CD44, PerCP-conjugated anti-CD3, PE-conjugated anti-CD8, FITC-conjugated anti-Granzyme A, anti-Granzyme B, anti-perforin and anti-IFN-γ were all purchased from BD Pharmingen. PE-conjugated anti-CD133 was purchased from eBiosciences. For cell surface staining, SMMC7721 and HepG2 cells were resuspended in 100μl staining buffer containing 10% FBS and put on ice for 20 min to block Fc receptors, then incubated with FITC-conjugated anti-CD44and PE-conjugated anti-CD133or isotype control for 30 min. The cells were then washed with 1ml ice-cold staining buffer for 2 times and centrifuged (300g) at 4°C for 5 min. The collected cells were suspended in 500μl staining buffer solution. PBMCs were incubated with SMMC7721 cells for 5 hours with brefeldin A at a final concentration of 10μg/ml, then collected and washed. As discussed above, PerCP-conjugated anti-CD3 and PE-conjugated anti-CD8 were used for surface staining. After that, cells were fixed and permeabilized, and then intracellular staining of FITC-conjugated anti-GranzymeA, Granzyme B, perforin and IFN-γ was performed. All samples after staining were evaluated using BD FACS Canto II with Diva software and analyzed using Flowjo 7.6.5.

### Cell apoptosis assay

Annexin V-PE and 7-AAD (BD Pharmingen) were used to measure the apoptotic levels of SMMC7721 and HepG2 cells. Cells transfected with B7-H4 siRNA plasmid or scramble siRNA plasmid were seeded in the six well cell culture plates at a density of 2 × 10^5^ cells per well and cultured for 48 hours. Adherent and floating cells were harvested, stained with Annexin V-PE/7-AAD and analyzed using flow cytometry.

### Analysis of degranulation of CD8^+^ T cells

Degranulation of CD8^+^ T cells was measured by CD107a mobilization assay. PBMCs were incubated with wide-type SMMC7721 cells or B7-H4-depleted SMMC7721 cells in RPMI media containing 10% fetal calf serum with FITC-conjugated anti-CD107a (BD Pharmingen) for 1 hour at 37°C in a 5% CO_2_ incubator, followed by an additional 5-hour incubation in the presence of the monensin. Cells were collected and washed at 1, 3, 5 hours after the addition of monensin, then stained with PerCP-conjugated anti-CD3 and PE-conjugated anti-CD8 and analyzed using flow cytometry.

### Transwell assay

In the transwell assay, HCC cells (SMMC7721 and HepG2 cells) were transfected with B7-H4 siRNA and the corresponding negative control cells were suspended with serum-free RPMI medium and then seeded in the upper chamber, 10% FBS contained medium was placed in the lower chamber. Then cells were incubated for 24 h and removed from the upper chamber surface of 8μm pores polycarbonate filter coated with 1mg/ml Matrigel (Corning, Inc.) to the lower chamber. The cells on the lower surface of the filter were fixed in methanol and stained with 0.1% crystal violet. The stained cells were washed three times with PBS, and counted in five random high power fields.

### Animal studies

Animal studies were performed and approved by the Animal Ethics Committee of the third hospital, Hebei Medical University. Negative control cells or cells stably transfected with B7-H4 shRNA were resuspended in PBS and subcutaneously implanted into the left and right flanks (5×10^6^ cells per flank) of 4-week-old male BALB/c nude mice (n=10). Tumor volumes were examined each week by measuring their length (a) and width (b) using a vernier caliper. The tumor volume (V) was calculated according to the formula with the equation, volume (mm^3^) = (length × width^2^)/^2^. The statistical significance between the tumor sizes in the control and B7-H4 shRNA-transfected groups was evaluated using Student's t-test.

### Statistical analysis

Statistical analysis was performed using SPSS 20.0 (IBM, USA). The Mann-Whitney U test, χ2 test, Pearson chi square test or Spearman rho test were performed for comparative statistical evaluations among the groups and for correlation analysis with histological and clinical parameters (age, gender, tumor stage, tumor grade, and postoperative survival). Survival periods were counted in months from the date of first visit to date of death or last follow-up before study closure. We used Kaplan-Meier method to estimate the OS for low and high levels of B7-H4 expression. Data were presented as mean ± standard deviation, and inter-group differences were assessed for significance using Student's t test. All statistics should be two-tailed, and the threshold of significance was set at *p*<0.05.

## SUPPLEMENTARY MATERIALS FIGURE AND TABLE


