# Supplementary material for: B7-H4 overexpression is essential for early hepatocellular carcinoma progression and recurrence
Source: Oncotarget. 2017 Sep 8;8(46):80878–88. doi: 10.18632/oncotarget.20718 (PMC5655246; doi:10.18632/oncotarget.20718)
Supplement: Supplementary file 1 [file oncotarget-08-80878-s001.pdf]

B7-H4 overexpression is essential for early hepatocellular carcinoma progression and recurrence

SUPPLEMENTARY MATERIALS

Supplementary Table 1: Univariable and multivariable analysis for of HCC survival

Characteristics	Univariate		Multivariate	
	**HR (95% CI)**	***p* value**	**HR (95% CI)**	***p* value**
Vascular Invasion	2.772 (1.158-5.228)	0.023	0.511 (0.107-1.566)	0.122
TNM stage	5.713 (1.786-11.003)	0.041	0.312 (0.064-1.021)	**0.021**
Metastasis	3.579 (1.299-9.862)	0.032	5.175 (1.735-19.661)	**0.032**
Recurrence	10.473 (3.121-52.326)	0.021	0.033 (0.003-0.326)	**0.017**
B7-H4 (expression)	0.411 (0.036-1.208)	0.026	0.177 (0.012-2.001)	0.155
B7-H4 (intensity)	4.132 (1.653-7.517)	0.019	0.256 (0.171-0.901)	**0.019**

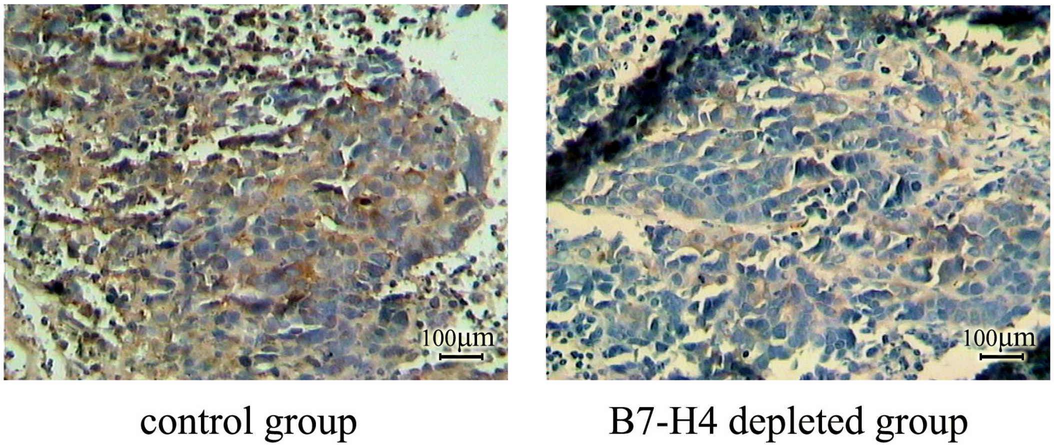

Supplementary Figure 1: Representative images of positive (left) and negative (right) B7-H4 expression in xenografted mouse model after treatment with negative control or B7-H4 depleted HCC cells.
